# New Therapeutic Agent against Arterial Thrombosis: An Iridium(III)-Derived Organometallic Compound

**DOI:** 10.3390/ijms18122616

**Published:** 2017-12-05

**Authors:** Chih-Wei Hsia, Marappan Velusamy, Jeng-Ting Tsao, Chih-Hsuan Hsia, Duen-Suey Chou, Thanasekaran Jayakumar, Lin-Wen Lee, Jiun-Yi Li, Joen-Rong Sheu

**Affiliations:** 1 Department of Pharmacology and Graduate Institute of Medical Sciences, College of Medicine, Taipei Medical University, 250 Wu-Hsing Street, Taipei 110, Taiwan; d119106003@tmu.edu.tw (C.-W.H.); mvelusamy@gmail.com (M.V.); p95421019@ntu.edu.tw (J.-T.T.); d119102013@tmu.edu.tw (C.-H.H.); fird@tmu.edu.tw (D.-S.C.); tjaya_2002@yahoo.co.in (T.J.); 2 Department of Chemistry, North Eastern Hill University, Shillong 793022, India; 3 Division of Allergy and Immunology, Department of Internal Medicine, Cathay General Hospital, Taipei 106, Taiwan; 4 Department of Microbiology and Immunology, Taipei Medical University, Taipei 110, Taiwan; lucie@tmu.edu.tw; 5 Department of Cardiovascular Surgery, Mackay Memorial Hospital, and Mackay Medical College, Taipei 104, Taiwan; jyl5891@gmail.com

**Keywords:** Ir(III)-derived complex, platelet activation, protein kinases, OH**·** free radical, bleeding time, pulmonary thromboembolism

## Abstract

Platelet activation plays a major role in cardio and cerebrovascular diseases, and cancer progression. Disruption of platelet activation represents an attractive therapeutic target for reducing the bidirectional cross talk between platelets and tumor cells. Platinum (Pt) compounds have been used for treating cancer. Hence, replacing Pt with iridium (Ir) is considered a potential alternative. We recently developed an Ir(III)-derived complex, [Ir(Cp*)1-(2-pyridyl)-3-(2-hydroxyphenyl)imidazo[1,5-a]pyridine Cl]BF_4_ (Ir-11), which exhibited strong antiplatelet activity; hence, we assessed the therapeutic potential of Ir-11 against arterial thrombosis. In collagen-activated platelets, Ir-11 inhibited platelet aggregation, adenosine triphosphate (ATP) release, intracellular Ca^2+^ mobilization, P-selectin expression, and OH**·** formation, as well as the phosphorylation of phospholipase Cγ2 (PLCγ2), protein kinase C (PKC), mitogen-activated protein kinases (MAPKs), and Akt. Neither the adenylate cyclase inhibitor nor the guanylate cyclase inhibitor reversed the Ir-11-mediated antiplatelet effects. In experimental mice, Ir-11 prolonged the bleeding time and reduced mortality associated with acute pulmonary thromboembolism. Ir-11 plays a crucial role by inhibiting platelet activation through the inhibition of the PLCγ2–PKC cascade, and the subsequent suppression of Akt and MAPK activation, ultimately inhibiting platelet aggregation. Therefore, Ir-11 can be considered a new therapeutic agent against either arterial thrombosis or the bidirectional cross talk between platelets and tumor cells.

## 1. Introduction

Intravascular thrombosis is a cause of various cardiovascular diseases (CVDs). The growth of thrombus inside the stent lumen is the outcome of platelet adhesion, and platelet activation followed by platelet aggregation. Thus, platelets play a crucial role in the pathogenesis of CVDs, including coronary artery disease and stroke [1]. Platelets are also critical for maintaining the integrity of the vascular system, and are the first-line defense against hemorrhage. During platelet activation, the release of several mediators (e.g., adenosine triphosphate (ATP) and thromboxane A_2_) occurs in conjunction with relative intracellular Ca^2+^ ([Ca^2+^]i) mobilization; these processes attract additional platelets toward the injured endothelium, and consequently cause thickening of the initial platelet monolayer. Finally, fibrinogen binds to its specific platelet receptor (integrin α_IIb_β_3_), thus completing the final common pathway for platelet aggregation. Platelet surface membrane contains glycoprotein IIb/IIIa receptors, receptors for thromboxane, adenosine diphosphate (ADP), thrombin, serotonin, epinephrine, histamine, and PAF [2]. Platelets are activated via high-affinity and low-affinity hypersensitivity receptors, which can induce Kounis hypersensitivity-associated thrombotic syndrome.

Platelet activation has also been associated with the key steps of cancer progression. Platelets have been proposed to affect malignancy development through a controlled process that triggers the pathobiology of cancer cells. Cancer cells interact with all the major components of the hemostatic system, including platelets. Platelets are involved in some critical steps of cancer metastasis, including regulation of tumor cell migration, invasion, and arrest within the vasculature [3,4]. The contents of platelets may be released into the peritumoral space following platelet activation, thus enhancing tumor cell extravasation and metastases [5]. Hence, a complex interaction between platelet-induced tumor growth and tumor-stimulated platelet activation occurs with the association of several machineries within the tumor microenvironment that augment metastasis.

Casing polymers, and metals establish an essential class of substances that can act as antigens. Apart from the well-known importance of nickel, chromium, and cobalt in triggering skin hypersensitivity, other metals such as aluminum, beryllium, copper, gold, iridium, mercury, palladium, platinum, rhodium, and titanium are developing as human body sensitizers. Iridium (Ir) is a noble and precious metal belonging to the platinum (Pt) group elements, which also consist of rare metals such as Pt, palladium, rhodium, ruthenium, and osmium. These metals have similar physical and chemical properties [6]. In nature, metallic Ir can be obtained from Pt ores. It is also obtained as a by-product of nickel mining and processing [7]. Various metal complexes have been identified as anticancer therapeutic agents; thus, an increasing amount of related research is available. Metal complexes provide a highly versatile platform for drug design. Metal ions have variable geometries and coordination numbers; hence, their chemical reactivity in terms of both kinetics (ligand exchange rates) and thermodynamics (such as metal–ligand bond strength and redox potentials) can be modified. Metals and their ligands pay crucial roles in biological activity. 

**Figure 1:**
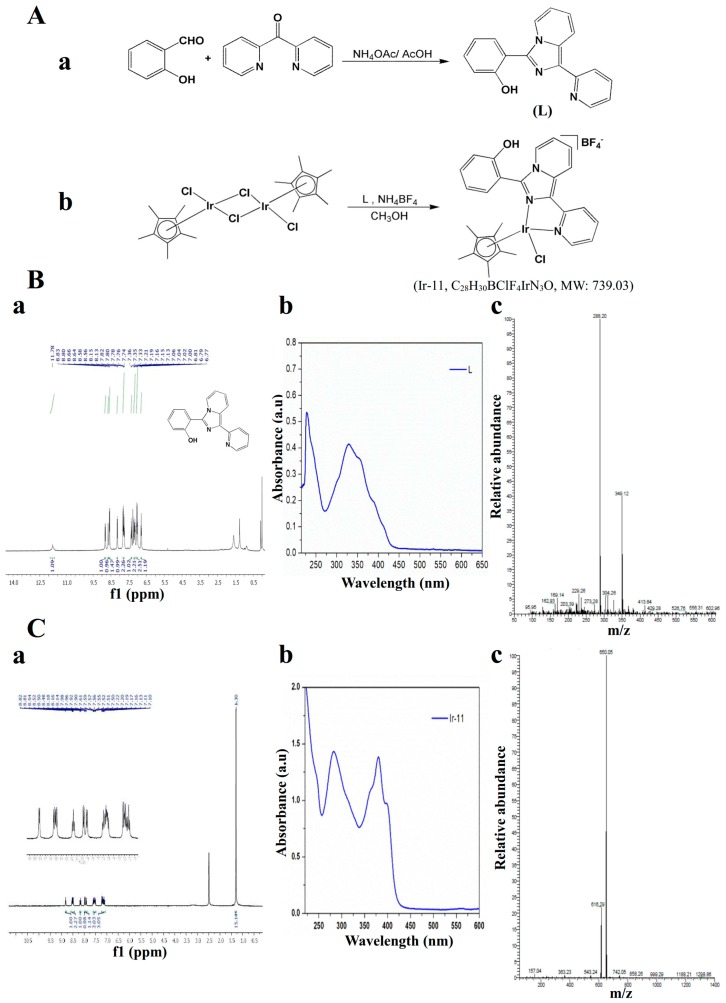
Chemical synthesis of Ir-11 compound. (**A**) Synthesis of (**a**) ligand (L) and (**b**) the complex [Ir(Cp*)(L)Cl]BF_4_ (Ir-11); (**B**) Spectral image of ligand 2-(1-pyridin-2-yl-imidazo[1,5-a]pyridin-3-yl)-phenol (**a**) nuclear magnetic resonance (NMR) (**b**) UV–vis absorption and (**c**) electrospray ionization mass spectrometry (ESI-MS); (**C**) Spectral image of Ir-11complex (**a**) NMR (**b**) UV–vis absorption and (**c**) ESI-MS.

Organometallic Ir(III) complexes are particularly promising. Currently, researchers are focusing on Ir(III) compounds because these compounds exhibit potential antitumor activity and low toxicity toward normal tissues [8,9]. Furthermore, Ir complexes exert potent antiangiogenic effects by activating distinct antiangiogenic signaling pathways [8]. Antiangiogenic therapy is considered a promising cancer treatment strategy. On the basis of these observations, we developed a new biologically active Ir(III) derivative, also referred to as Ir-11 (Figure 1). Although in vitro and in vivo pharmacological studies have demonstrated that Ir-based compounds exhibit potent anticancer activity, to date, no study has investigated their effects on platelet activation. Preliminary studies have reported strong activity of Ir-11 toward human platelets. Thus, we further examined the characteristics and functional activity of Ir-11 in platelet activation ex vivo and in vivo. The present study confirms the development of a new class of Ir-based antiplatelet agent.

## 2. Results

### 2.1. Inhibitory Effects of Ir-11 on Aggregation of Washed Human Platelets

**Figure 2:**
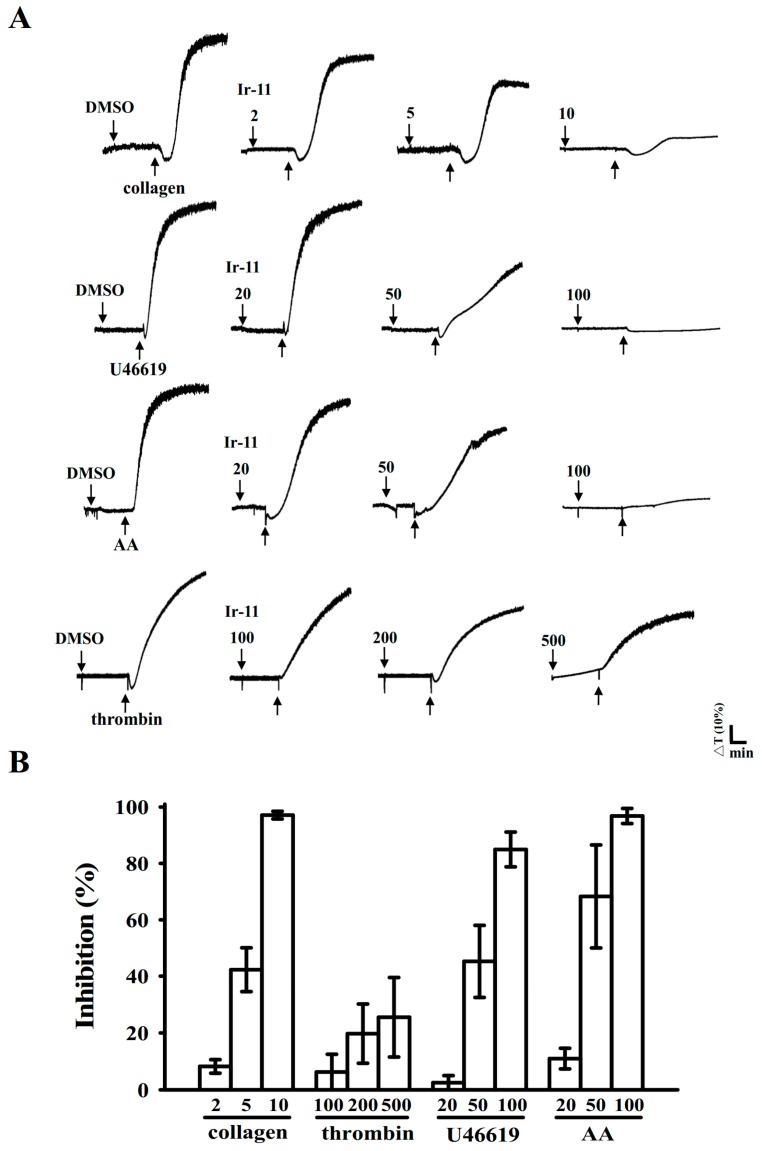
Comparison of the relative inhibitory activity of Ir-11 against platelet aggregation stimulated by various agonists in washed human platelets. (**A**) Washed human platelets (3.6 × 10^8^ cells/mL) were preincubated with the solvent control 0.1% DMSO or various concentrations of Ir-11 (2–500 µM) and subsequently treated with collagen (1 µg/mL), thrombin (0.01 U/mL), U46619 (1 µM), and AA (120 µM) to stimulate platelet aggregation; (**B**) Concentration–response histograms of Ir-11 against platelet aggregation stimulated by agonists. Data are presented as means ± standard error of the mean (*n* = 4).

Ir-11 (2–10 µM; Figure 2) strongly inhibited aggregation of collagen-stimulated (1 µg/mL) human platelets in a concentration-dependent manner. Furthermore, Ir-11 (20–100 µM) exhibited moderate activity against stimulation by arachidonic acid (AA, 120 µM) or U46619 (1 µM), a prostaglandin endoperoxide; however, Ir-11 (100–500 µM) exhibited relatively low inhibition of platelet aggregation that was stimulated using thrombin (0.01 U/mL) (Figure 2). The 50% inhibitory concentration (IC_50_) of Ir-11 for collagen-stimulated aggregation was approximately 6 µM. Therefore, in the subsequent experiments, the IC_50_ (6 µM) and maximal concentration (10 µM) of Ir-11 were used for exploring the possible mechanisms of action of Ir-11 on human platelets. In addition, the solvent control (0.1% dimethyl sulfoxide, DMSO) did not significantly affect platelet aggregation (Figure 2A).

### 2.2. Regulatory Role of Ir-11 in Adenosine Triphosphate (ATP) Release, Relative [Ca^2+^]i Mobilization, Surface P-Selectin Expression, Cytotoxicity, and Cyclic Nucleotide Formation in Washed Human Platelets 

**Figure 3:**
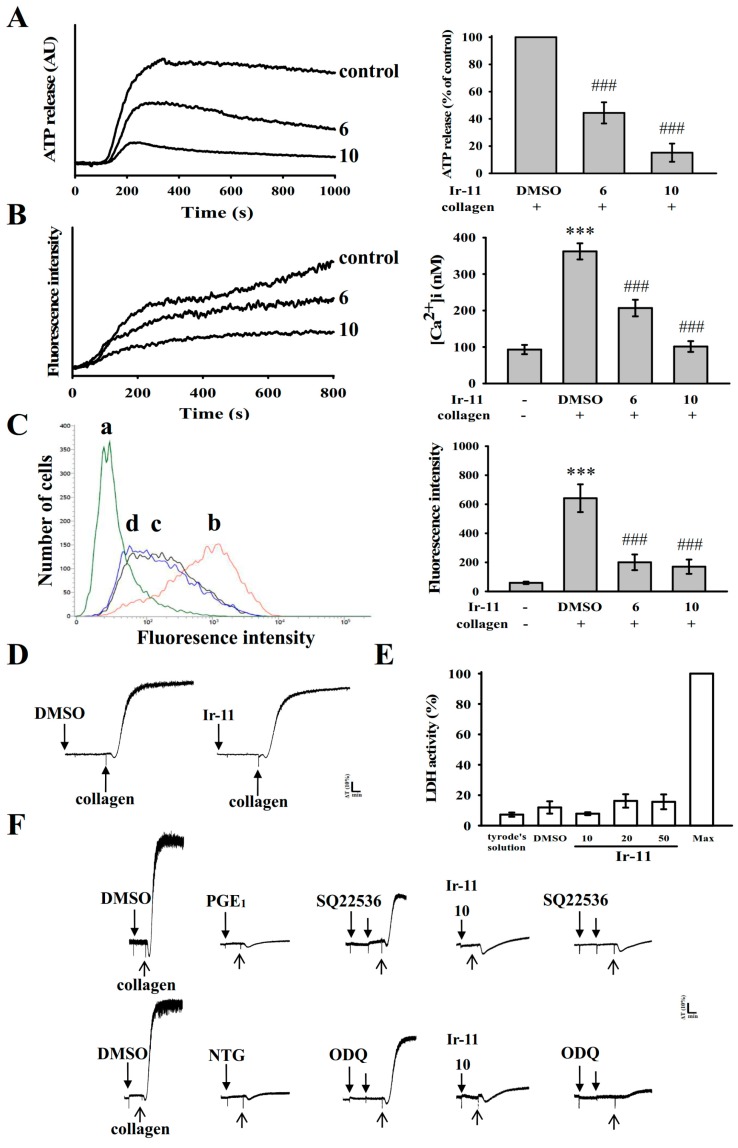
Effect of Ir-11 on ATP release, relative [Ca^2+^]i mobilization, surface P-selectin expression, cytotoxicity, LDH release, and cyclic nucleotide formation in human platelets. Washed platelets (3.6 × 10^8^ cells/mL) were preincubated with the solvent control (0.1% DMSO), Ir-11 (6 and 10 µM), or fluorescein isothiocyanate (FITC)–P-selectin (2 µg/mL), and collagen (1 μg/mL) was then added to trigger either (**A**) ATP release (AU; arbitrary unit), (**B**) relative [Ca^2+^]i mobilization, or (**C**) surface P-selectin expression (a) Tyrode’s solution (resting control), (b) 0.1% DMSO, (c) 6 µM Ir-11, or (d) 10 µM Ir-11. The corresponding statistical data are shown in the right panel of each figure (**A**–**C**); (**D**) Washed platelets were preincubated with the solvent control (0.1% DMSO) or Ir-11 (50 µM) for 10 min and subsequently washed two times with Tyrode solution; collagen (1 μg/mL) was then added to trigger platelet aggregation; (**E**) Washed platelets were preincubated with the solvent control (0.1% DMSO) or Ir-11 (10, 20, and 50 µM) for 20 min, and a 10 µL aliquot of the supernatant was deposited on a Fuji Dri-Chem slide LDH-PIII; (**F**) For other experiments, washed platelets were preincubated with PGE_1_ (1 µM), NTG (10 µM), or Ir-11 (10 µM) with or without SQ22536 (100 µM) or ODQ (10 μM), and were subsequently treated with collagen (1 µg/mL) to induce platelet aggregation. Data are presented as means ± standard error of the means (*n* = 4). Profiles in (**D**,**F**) are representative of four independent experiments. *** *p* < 0.001 compared with the resting control; ^###^ *p* < 0.001, compared with the DMSO-treated group.

Platelet activation is associated with the release of granular contents (e.g., ATP and Ca^2+^from the dense granules, and P-selectin from the α-granules), resulting in substantial platelet activation. In the present study, Ir-11 (6 and 10 µM) markedly reduced both ATP release (Figure 3A) and relative [Ca^2+^]i mobilization (resting control, 93.0 ± 12.7 nM; collagen-stimulated, 362.3 ± 22.1 nM; 6 µM, 206.9 ± 22.7 nM; and 10 µM, 101.3 ± 14.8 nM; *n* = 4, Figure 3B) in platelets stimulated by collagen (1 µg/mL). In resting platelets, P-selectin is located on the inner wall of the α-granules. Platelet activation exposes the inner walls of the granules to the outside of the cell [10]. Treatment with Ir-11 markedly reduced collagen-induced surface P-selectin expression (resting control, 59.3 ± 8.5; collagen-activated, 641.8 ± 95.7; 6 μM, 200.5 ± 54.1; 10 μM, 170.3 ± 49.2; *n* = 4, Figure 3C). The corresponding statistical data are presented in the right panels of Figure 3A–C.

The aggregation curves of platelets preincubated with Ir-11 (50 µM) for 10 min and subsequently washed two times with Tyrode solution were not significantly different from those of platelets preincubated with the solvent control (0.1% DMSO) under equivalent conditions (Figure 3D); this observation preliminarily indicated that the effects of Ir-11 on platelet aggregation are reversible and noncytotoxic. The lactate dehydrogenase (LDH) detection results revealed that Ir-11 (10, 20, and 50 µM) incubated with platelets for 20 min did not significantly increase LDH activity in the platelets or exert cytotoxic effects on them (Figure 3E), thus demonstrating that Ir-11 does not affect platelet permeability or induce platelet cytolysis. Furthermore, SQ22536 (9-(tetrahydro-2-furanyl)-9H-purin-6-amine, 100 μM), an adenylate cyclase inhibitor, and ODQ (1H-[1,2,4] oxadiazolo [4,3-a]quinoxalin-1-one, 10 µM), a guanylate cyclase inhibitor, significantly reversed the prostaglandin E_1_ (PGE_1_)-mediated (1 µM) or nitroglycerin (NTG)-mediated (10 μM) inhibition of platelet aggregation stimulated by collagen (Figure 3F). Neither SQ22536 nor ODQ significantly reversed Ir-11-mediated antiplatelet activity stimulated by collagen (Figure 3F).

### 2.3. Effect of Ir-11 on the Phospholipase Cγ2/Protein Kinase C (PLCγ2/PKC) Cascade and Mitogen-Activated Protein Kinase (MAPK) and Akt Activation 

**Figure 4:**
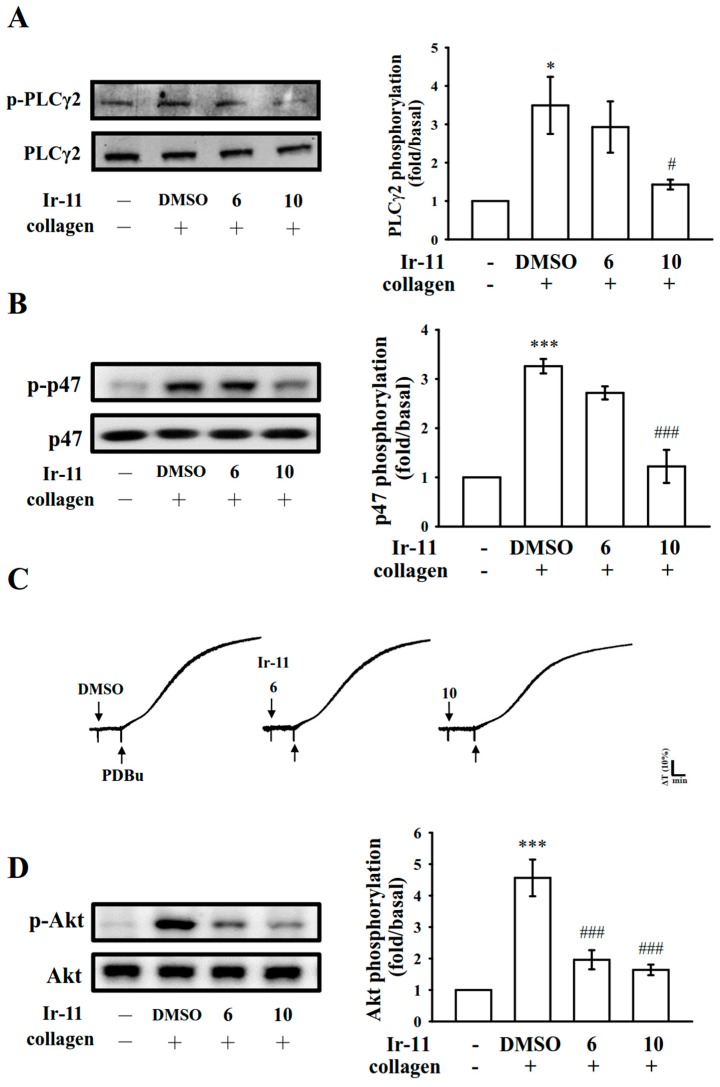
Inhibitory effects of Ir-11 on PLCγ2, PKC, and Akt activation in platelets. Washed platelets were preincubated with the solvent control (0.1% DMSO) or Ir-11 (6 and 10 µM), and subsequently treated with collagen (1 µg/mL) or PDBu (150 nM) to induce (**A**) PLCγ2 and (**B**) PKC activation (p47, pleckstrin phosphorylation), (**C**) platelet aggregation, and (**D**) Akt phosphorylation. Platelets were collected, and their subcellular extracts were analyzed to determine the levels of protein phosphorylation. Data are presented as means ± standard error of the means (*n* = 4). * *p* < 0.05 and *** *p* < 0.001, compared with the resting control; ^#^ *p* < 0.05 and ^###^ *p* < 0.001, compared with the DMSO-treated group. The profiles in (**C**) are representative of four independent experiments.

**Figure 5:**
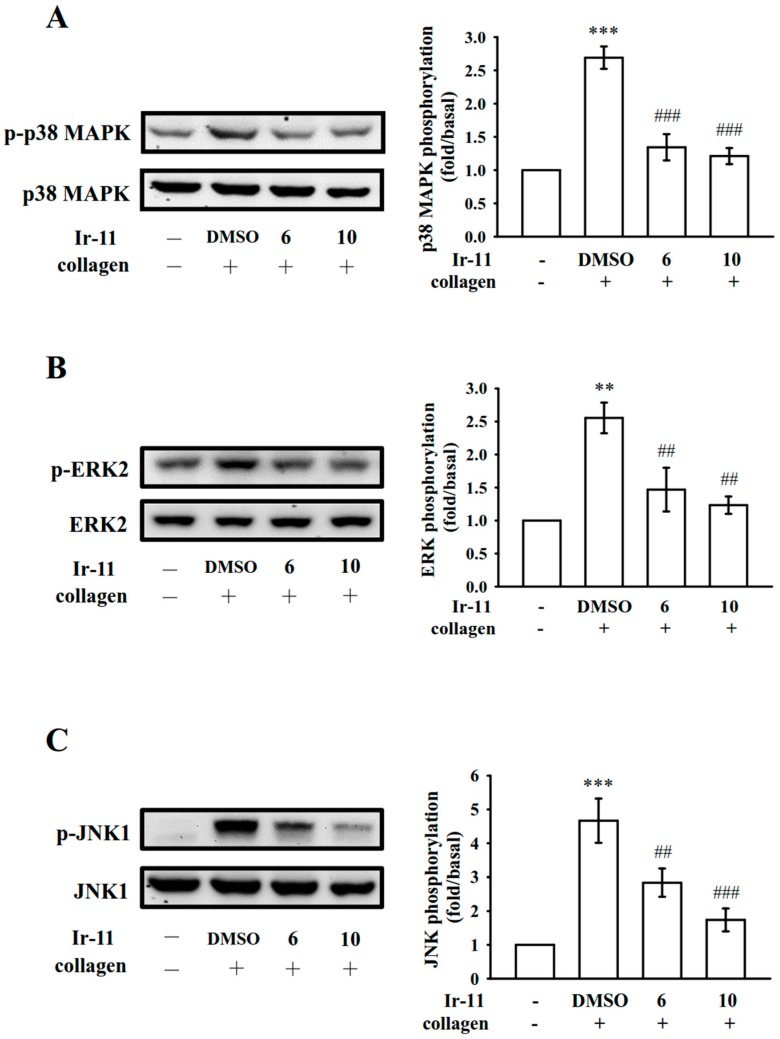
Effects of Ir-11 on p38 MAPK, ERK2, and JNK1 phosphorylation in collagen-activated platelets. Washed platelets were preincubated with the solvent control (0.1% DMSO) or Ir-11 (6 and 10 µM) and subsequently treated with collagen (1 µg/mL) to trigger (**A**) p38 MAPK, (**B**) ERK2, and (**C**) JNK1 activation. Platelets were collected, and their subcellular extracts were analyzed to determine the levels of protein phosphorylation. Data are presented as means ± standard error of the means (*n* = 4). ** *p* < 0.01 and *** *p* < 0.001, compared with the resting control; ^##^ *p* < 0.01 and ^###^ *p* < 0.001, compared with the DMSO-treated group.

PLCs hydrolyze phosphatidylinositol 4,5-bisphosphate to generate the secondary messengers inositol 1,4,5-trisphosphate (IP_3_) and diacylglycerol (DAG). IP_3_ triggers relative [Ca^2+^]i mobilization and DAG activates PKC, yielding an approximately 47 kDa protein that is predominantly phosphorylated (p47 protein; pleckstrin) and causes ATP release [11]. As stated previously, Ir-11 markedly reduced relative [Ca^2+^]i mobilization (Figure 3B). We further examined the effect of Ir-11 on the phosphorylation of the PLCγ2-PKC signaling cascade. As shown in Figure 4A,B, Ir-11 (10 µM) markedly reduced both PLCγ2 phosphorylation and PKC activation (p47 phosphorylation) in collagen-activated platelets. Nonetheless, Ir-11 (6 and 10 µM) did not significantly affect platelet aggregation stimulated by 150 nM PDBu, a well-known PKC activator (Figure 4C), thus indicating that although Ir-11 did not directly disturb PKC activation, it may have interfered with the upstream regulators of PKC, such as PLCγ2. Additionally, Akt is a serine/threonine-specific protein kinase that plays a key role in multiple cellular processes, such as platelet activation, cell proliferation, apoptosis, and cell migration [12]. Ir-11 (6 and 10 µM) also markedly inhibited collagen-induced Akt phosphorylation (Figure 4D). To further analyze the inhibitory mechanisms of Ir-11, several signaling molecules associated with the mitogen-activated protein kinases (MAPKs) were evaluated. The major MAPK kinase, including p38 MAPK, extracellular signal–regulated kinases (ERKs), and c-Jun N-terminal kinases (JNKs), regulate cellular responses in eukaryotic organisms through cell proliferation, migration, differentiation, and apoptosis. ERK2, JNK1, and p38 MAPK have been identified in platelets [13]. As shown in Figure 5A–C, Ir-11 (6 and 10 µM) inhibited collagen-stimulated phosphorylation of all these proteins in a concentration-dependent manner.

### 2.4. Assessment of OH·-Scavenging Activity of Ir-11 through ESR Spectrometry 

**Figure 6:**
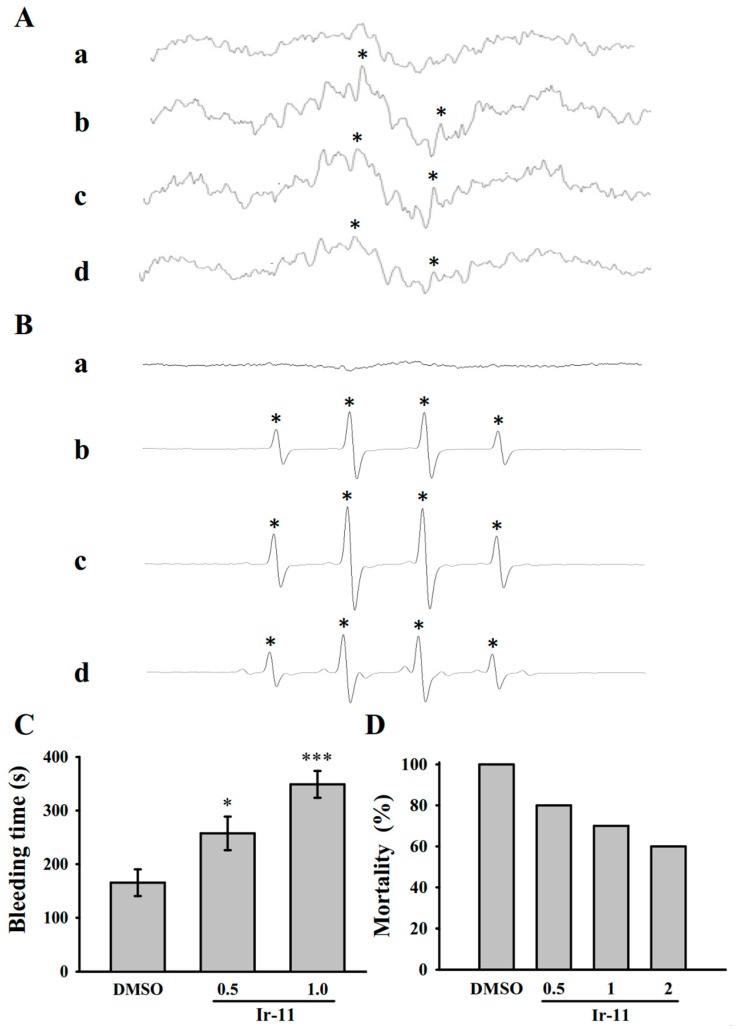
Regulatory activities of Ir-11 on OH**·** formation in platelet suspensions or the Fenton reaction solution, and bleeding time in the tail vein as well as acute pulmonary thromboembolism in experimental mice. (**A**) Washed platelets or (**B**) the Fenton reaction solution was preincubated with Tyrode solution (**a**, resting control), (**b**) 0.1% DMSO, or Ir-11 at (**c**) 6 µM or (**d**) 10 µM. Collagen (1 µg/mL) was then added for the ESR experiments as described in **Materials and Methods**. Profiles are representative of four independent experiments, and an asterisk (*) indicates OH**·** formation; (**C**) The bleeding time was measured through transection of the mouse tail after 30 min of administering either 0.1% DMSO, 0.5 mg/kg, or 1.0 mg/kg Ir-11 intraperitoneally (all in 50 µL); (**D**) For acute pulmonary thrombosis study, 0.1% DMSO or Ir-11 in various doses (0.5, 1, and 2 mg/kg) (all in 50 µL) was administered intraperitoneally to the mice, and ADP (0.7 mg/g) was then injected through the tail vein. Data are presented as means ± standard error of the means (C, *n* = 8; D, *n* = 10). * *p* < 0.05 and *** *p* < 0.001, compared with the DMSO (0.1%)-treated group.

An ESR signal indicative of OH**·** radical formation was observed in both collagen-stimulated platelet suspensions (Figure 6(Ab)) and the Fenton reaction solution (cell-free system; Figure 6(Bb)) compared with the resting control (Figure 6(Aa,Ba)). Treatment with Ir-11 (6 and 10 μM) considerably reduced the OH**·** signals in the collagen-stimulated platelet suspensions (Figure 6(Ac,d)) but not in the Fenton reaction solution (Figure 6(Bc,d)), suggesting that Ir-11 reduced intracellular OH**·** radical formation, but it did not exhibit this effect in a cell-free system.

### 2.5. Crucial Roles of Ir-11 in Bleeding Time and Adenosine Diphosphate (ADP)-Induced Acute Pulmonary Thromboembolism In Vivo 

In the tail transection model of mice, after 30 min of administering 0.5 or 1.0 mg/kg Ir-11 intraperitoneally, the bleeding times recorded were 165.4 ± 24.9 s (0.1% DMSO-treated group; *n* = 8), 257.4 ± 31.3 s (group treated with Ir-11, 0.5 mg/kg; *n* = 8), and 348.9 ± 25.1 s (group treated with Ir-11, 1.0 mg/kg; *n* = 8) (Figure 6C). Each mouse was monitored for 10 min after the bleeding stopped, for detection of any re-bleeding. We also investigated the therapeutic effects of Ir-11 in preventing acute pulmonary embolism death in mice, as shown in Figure 6D. The results indicated that Ir-11 significantly lowered mortality in mice challenged with ADP (0.7 mg/g), and treatment with Ir-11 at 0.5, 1.0, and 2.0 mg/kg considerably reduced mortality from control (DMSO-treated, 100%) to 80%, 70%, and 60% (*n* = 10), respectively. 

## 3. Discussion

In addition to the regulation of hemostasis and coagulation, platelets play a crucial role in potentiating tumor cell growth and metastasis [14]. The activation of platelets is associated with the thrombotic events in patients with cancer [15]. Chemotherapeutics may amplify this effect and stimulate vascular thromboembolic events (VTEs) by aggravating endothelial cell damage, augmenting platelet aggregation, aggregating oxidative damage, and consequently leading vascular toxicity [16]. Among the Pt-based chemotherapy agents, cisplatin is associated with a high incidence of treatment-related VTEs [17]. Gemcitabine, combined with a Pt-based agent, is associated with increased thrombotic and vascular side effects [18,19]. Therefore, researchers are focusing on the development of new metal-based agents for inhibiting platelet activation to treat vascular diseases, reduce toxic side effects, and overcome Pt resistance. Notably, this study provides preliminary evidence demonstrating that Ir-11, belonging to a novel class of synthetic Ir(III)-derived compound, exhibits powerful antiplatelet activity ex vivo and in vivo. 

Platelets adhere to the subendothelial matrix (i.e., collagen), thus altering their shape and releasing granular contents (e.g., ATP, Ca^2+^, and P-selectin). P-selectin is an adhesion molecule stored in the α-granules of platelets, and it is expressed on the platelet surface membrane upon activation. Subsequently, it is expressed on the external membrane through membrane flipping. P-selectin mediates the initial formation of platelet aggregates and facilitates the formation of large platelet aggregates [20]. Several agonists, such as collagen, thrombin, and AA, mobilize [Ca^2+^]i to phosphorylate the Ca^2+^/calmodulin-dependent myosin light chain (20 kDa), which is involved in the secretion of granule contents, such as serotonin and ATP [21], as well as platelet aggregation. Therefore, the inhibition of relative [Ca^2+^]i mobilization and ATP production are crucial for evaluating the antiplatelet effects of a compound. In the present study, Ir-11 inhibited platelet aggregation to different degrees, depending on the agonist used, indicating that Ir-11 did not act as the specific individual receptor of these agonists. Therefore, Ir-11 probably exerts its inhibitory effects on stimulated platelets through a common signaling cascade. 

Platelet activation by agonists, such as collagen, substantially alters PLC activation. PLC activation results in IP_3_ and DAG production, which activates PKC and consequently induces p47 phosphorylation [11]. PKC activation triggers particular responses, facilitating the transmission of specific activating signals in distinct cellular compartments. The PLCγ family consists of the isozymes PLCγ1 and PLCγ2; PLCγ2 is involved in collagen-dependent signaling in human platelets [22]. Ir-11 considerably reduced collagen-induced PLCγ2-PKC activation; however, Ir-11 did not exert direct effects on PKC activation because it did not inhibit PDBu-induced platelet aggregation, suggesting that the Ir-11-mediated inhibition of platelet activation involves PLCγ2 downstream signaling. This result also explains why Ir-11 was more efficacious in inhibiting platelet aggregation induced by collagen, than that induced by thrombin, U46619, and AA.

Human platelet activation is inhibited through intracellular cyclic-AMP- and cyclic-GMP-mediated pathways, and cyclic nucleotides are crucial modulators of platelet activation [23]. At elevated levels, cyclic nucleotides inhibit most platelet responses and reduce the [Ca^2+^]i level by mediating Ca^2+^ uptake by the dense tubular system; thus, the cyclic nucleotides suppress PLC and PKC activation [22]. Therefore, cyclic AMP and cyclic GMP synergistically inhibit platelet activation. In the present study, neither SQ22536 nor ODQ significantly reversed the Ir-11-mediated inhibition of collagen-induced platelet aggregation. Therefore, the Ir-11-mediated mechanisms are independent of increasing cyclic nucleotide formation in platelets.

MAPKs are activated by specific MAPK kinases (MEKs); specifically, MEK1/2, MEK3/6, and MEK4/7 activate ERKs, p38 MAPK, and JNKs, respectively [24]. Cytosolic phospholipase A_2_ (cPLA_2_) is a substrate of p38 MAPK activity induced by various agonists, such as von Willebrand factor (vWF) and thrombin [25]. Therefore, p38 MAPK is essential for cPLA_2_ stimulation and AA release [26]. We observed that SB203580, a p38 MAPK inhibitor, inhibited collagen-induced platelet aggregation substantially [27]. ERK activation is involved in platelet aggregation requiring prior ATP release, which triggers P_2_X_1_-mediated Ca^2+^ influx and activates ERKs, thereby increasing the phosphorylation of myosin light chain kinase [24]. JNK1 is the most recently identified MAPK in platelets, and therefore, its activation or role is poorly established. It is activated by several agonists such as thrombin, vWF, collagen, and ADP [28]. In addition, a study demonstrated that JNK^−/−^ platelets are associated with an increased bleeding time, decreased integrin α_IIb_β_3_ activation, and severe granule secretion impairment [25]. In accordance with these findings, the present results demonstrated that Ir-11 markedly inhibits the collagen-induced phosphorylation of these three MAPKs. 

Akt is a downstream effector of phosphoinositide 3 (PI3)-kinase. Akt-knockout mice have been reported to exhibit defects in agonist-induced platelet activation, suggesting that Akt regulates platelet activation, and that such regulation potentially has consequences concerning thrombosis [12,13]. Three mammalian Akt isoforms exist, namely Akt 1, 2, and 3. The first two isoforms were detected in human platelets [29]. Studies using Akt inhibitors in human platelet activation have typically reported similar roles for Akt 1 and 2. Consequently, protein kinases involved in Akt activation, chiefly PI3-kinase β, may be suitable targets for the production of antithrombotic therapeutics agents. In our previous study, we found that both PI3-kinase/Akt and MAPKs (e.g., p38 MAPK) are mutually activated as the upstream regulators of PKC in activated platelets [30]. 

Reactive oxygen species produced through platelet activation (i.e., H_2_O_2_ and OH**·**) might affect cells that they contact, such as endothelial cells, thereby enhancing platelet reactivity during thrombus formation. Free radicals upsurge [Ca^2+^]i levels during the early stage of platelet activation, and PKC plays role in the receptor-mediated production of free radicals in platelets [31]. In addition, H_2_O_2_ produced by platelets is converted into OH**·,** because platelet aggregation is inhibited by OH**·** scavengers [31]. Our ESR spectrometry results provide direct evidence that Ir-11 significantly reduced OH**·** formation in collagen-stimulated platelet suspensions.

In studies on acute pulmonary thromboembolism, platelet aggregation is intimately involved in experimental thrombosis, and Ir-11 effectively prevented ADP-induced thromboembolic death, as expected. Alternatively, we found that ADP induced mortality in mice was not able to reduce by heparin (1.5 U/g) treatment [32]. These data are consistent with the fact that platelet aggregation is a more crucial factor in inducing thromboembolism in rat animal model than fibrin formation. Furthermore, prolongation of hemostatic platelet plug formation (bleeding time) was observed in Ir-11-treated experimental mice. A cautious bleeding time analysis suggested that the elongation of bleeding time in humans does not predict the risk of hemorrhage or surgical bleeding, thereby questioning the rationale behind its use in the clinical evaluation of antiplatelet compounds [33]. 

## 4. Materials and Methods

### 4.1. Chemicals and Reagents

Thrombin, collagen, arachidonic acid (AA), luciferin–luciferase, U46619, phorbol 12, 13-dibutyrate (PDBu), nitroglycerin (NTG), heparin, prostaglandin E_1_ (PGE_1_), 5,5-dimethyl-1-pyrroline N-oxide (DMPO), SQ22536, ODQ, and bovine serum albumin (BSA) were purchased from Sigma (St. Louis, MO, USA). Fura-2AM was obtained from Molecular Probes (Eugene, OR, USA). An anti-phospho-p38 mitogen-activated protein kinase (MAPK) Ser^182^ monoclonal antibody (mAb) was purchased from Santa Cruz Biotechnology (Santa Cruz, CA, USA). Anti-p38 MAPK, anti-phospho-c-Jun N-terminal kinase (JNK) (Thr^183^/Tyr^185^), and anti-p44/42 extracellular signal-regulated kinase (ERK) mAbs, as well as anti-phospholipase Cγ2 (PLCγ2), anti-phospho (Tyr^759^) PLCγ2, anti-phospho-(Ser) protein kinase C (PKC) substrate (pleckstrin; p-p47), anti-JNK, and anti-phospho-p44/p42 ERK (Thr^202^/Tyr^204^) polyclonal antibodies (pAbs) were purchased from Cell Signaling (Beverly, MA, USA). Anti-phospho-protein kinase B (Akt) (Ser^473^) and anti-Akt mAbs were purchased from Biovision (Mountain View, CA, USA). An anti-pleckstrin (p47) pAb was purchased from GeneTex (Irvine, CA, USA). Hybond-P polyvinylidene fluoride (PVDF) membrane, enhanced chemiluminescence Western blotting detection reagent, horseradish peroxidase (HRP)-conjugated donkey anti-rabbit immunoglobulin G (IgG), and sheep anti-mouse IgG were purchased from Amersham (Buckinghamshire, UK). Fluorescein isothiocyanate (FITC) anti-human CD42P (P-selectin) mAb was obtained from BioLegend (San Diego, CA, USA). 

### 4.2. Synthesis of 1-(2-Pyridyl)-3-(2-hydroxyphenyl)imidazo[1,5-a]pyridine (L)

A mixture of di-pyridin-2-yl-methanone (0.92 g, 5 mM), 2-hydroxybenzaldehyde (1.22 g, 10 mM), and ammonium acetate (1.93 g, 25 mM) in 30 mL of glacial acetic acid was refluxed for 24 h under a nitrogen atmosphere by using an oil bath. After completion of the reaction, the mixture was cooled to ambient temperature and poured into a beaker containing deionized water. The precipitate that formed was then filtered, washed in an excess of water, and dried. The solid obtained was further purified through column chromatography on silica gel using hexane/ethyl acetate (3:1) as an eluent. An off-white solid was finally obtained. Yield: 66%; m.p. 184–190 °C; ^1^H NMR (400 MHz, CDCl_3_) δ 11.78 (bs, 1H), 8.83–8.80 (d, 1H, *J* = 12 Hz), 8.66–8.64 (d, 1H, *J* = 8 Hz), 8.58–8.56 (d, 1H, *J* = 8 Hz), 8.15–8.13 (d, 1H, *J* = 8 Hz), 7.82–7.74 (m, 2H), 7.37–7.33 (t, 1H, *J* = 8 Hz), 7.21–7.19 (d, 1H, *J* = 8 Hz), 7.16–7.13 (t, 1H, *J* = 6 Hz), 7.06–7.0 (m, 2H), 6.81–6.77 (t, 1H, *J* = 8 Hz); UV–vis: λ_abs_, nm: 227, 298, 387(sh); ESI-MS (*m*/*z*) 288.20 [M^+^ + H] (Figure 1A).

### 4.3. Synthesis of [Ir(Cp*)(L)Cl]BF_4_] (Ir-11) 

A suspension of L (0.11 g, 0.4 mM) and [Ir(Cp*)(Cl)_2_]_2_ dimer (0.16 g, 0.2 mM) in 10 mL of methanol was stirred for 2 h. NH_4_BF_4_ was added, and the mixture was stirred overnight. The resulting orange solution was evaporated; the precipitate formed was redissolved in dichloromethane and filtered. Finally, the filtrate was evaporated and washed with diethyl ether, and it yielded an orange solid. Yield: 89%; ^1^H NMR (400 MHz, dimethyl sulfoxide [DMSO]-*d*_6_) δ 8.82–8.81 (d, 1H, *J* = 4 Hz), 8.54–8.48 (m, 2H), 8.16–8.14 (t, 1H, *J* = 4 Hz), 7.98–7.90 (m, 2H), 7.61–7.50 (m, 3H), 7.22–7.10 (m, 3H), 1.30 (s, 15H); UV–vis, λ_abs_, nm (ε, M^−1^ cm^−1^): 239 (2624), 282 (2856), 308 (2082), 358 (2102), 379 (2775), 395 (1933); ESI-MS (*m*/*z*) 650.05 [M^+^-BF_4_^−^] (Figure 1B).

### 4.4. Platelet Aggregation

This study was approved by the Institutional Review Board of Taipei Medical University (TMU-JIRB-N201612050, 20 January 2017), and it conformed to the directives of the Declaration of Helsinki. All human volunteers involved in this study provided informed consent. Human platelet suspensions were prepared as described previously [34]. Blood samples were collected from adult human volunteers who had not taken any drugs or other substances for at least 14 days before collection; the collected blood samples were mixed with an acid–citrate–dextrose solution. After centrifugation, platelet-rich plasma (PRP) was mixed with 0.5 μM PGE_1_ and 6.4 IU/mL heparin. Tyrode solution comprising 3.5 mg/mL BSA was used to prepare the final suspension of washed human platelets. The final Ca^2+^ concentration in the Tyrode solution was 1 mM. A platelet aggregation study was conducted using a lumiaggregometer (Payton Associates, Scarborough, ON, Canada), as described previously [33]. An isovolumetric solvent control (0.1% DMSO) or Ir-11 was preincubated with platelet suspensions (3.6 × 10^8^ cells/mL) for 3 min before the addition of agonists (i.e., collagen). The extent of platelet aggregation was calculated as the percentage compared with individual control (without Ir-11) expressed in light transmission units, after the reaction proceeded for 6 min. For an ATP release assay, 20 µL of luciferin–luciferase was added 1 min before the addition of the collagen (1 µg/mL), and the amount of ATP released was compared with that released by the control (without Ir-11).

### 4.5. Measurement of Relative [Ca^2+^]i Mobilization by Using Fura-2AM Fluorescence 

The relative [Ca^2+^]i concentration was determined using Fura-2AM as described previously [34]. Briefly, citrated whole blood was centrifuged at 120× *g* for 10 min, and the PRP was collected and incubated with Fura-2AM (5 µM) for 1 h. Human platelets were prepared as described in the preceding section. The Fura-2AM-loaded platelets were preincubated with various concentrations of Ir-11 (6 and 10 µM) in the presence of 1 mM CaCl_2_ and then stimulated with collagen (1 µg/mL). The Fura-2 fluorescence was measured using a spectrofluorometer (Hitachi FL Spectrophotometer F-4500, Tokyo, Japan) at excitation wavelengths of 340 and 380 nm, and an emission wavelength of 510 nm.

### 4.6. Detection of Lactate Dehydrogenase

Washed platelets (3.6 × 10^8^ cells/mL) were preincubated with the solvent control (0.1% DMSO) or Ir-11 (10–50 μM) for 20 min at 37 °C. An aliquot of the supernatant (10 µL) was deposited on a Fuji Dri-Chem slide LDH-PIII (Fuji, Tokyo, Japan), and the absorbance wavelength was read at 540 nm using a UV–vis spectrophotometer (UV–160; Shimadzu, Japan). A maximal value of lactate dehydrogenase (LDH) was recorded in the sonicated platelets (Max).

### 4.7. Flow Cytometric Analysis of Surface P-Selectin Expression

Washed platelets were prepared as described in the preceding section, and the aliquots of platelet suspensions (3.6 × 10^8^ cells/mL) were preincubated with the solvent control (0.1% DMSO) or Ir-11 (6 and 10 µM) and FITC-P-selectin (2 µg/mL) for 3 min, and collagen (1 µg/mL) was added to trigger platelet activation. The suspensions were then assayed for fluorescein-labeled platelets by using a flow cytometer (FACScan System, Becton Dickinson, San Jose, CA, USA). Fifty thousand platelets/experimental group were used to collected data. To confirm reproducibility, all experiments repeated at least four times.

### 4.8. Immunoblotting of Protein Phosphorylation 

Washed platelets (1.2 × 10^9^ cells/mL) were preincubated with the solvent control (0.1% DMSO) or Ir-11 (6 and 10 µM) for 3 min. Subsequently, collagen (1 µg/mL) was added to stimulate platelet activation. After the reaction was stopped, the platelets were directly resuspended in 200 μL of lysis buffer. Samples comprising 80 μg of protein were separated through 12% sodium dodecyl sulfate gel electrophoresis, and the proteins were electrotransferred to PVDF membranes using a Bio-Rad semidry transfer unit (Bio-Rad, Hercules, CA, USA). The blots were then blocked by treating them with Tris-buffered saline in Tween 20 (TBST; 10 mM Tris-base, 100 mM NaCl, and 0.01% Tween 20) containing 5% BSA for 1 h, and were probed with various primary antibodies. The membranes were incubated for 1 h with HRP-conjugated anti-mouse IgG or anti-rabbit IgG (diluted 1:3000 in TBST). An enhanced chemiluminescence system was used to detect immunoreactive bands, and their optical density was quantified using Bio-profil Biolight (version V2000.01; Vilber Lourmat, Marne-la-Vallée, France). 

### 4.9. Measurement of OH· Formation in Either Platelet Suspensions or the Fenton Reaction Solution through Electron Spin Resonance Spectrometry

Electron spin resonance (ESR) spectrometry was performed using a Bruker EMX ESR spectrometer (Bruker, Billerica, MA, USA) as described previously [35]. Suspensions of washed platelets (3.6 × 10^8^ cells/mL) were preincubated with 0.1% DMSO or Ir-11 (6 and 10 μM) for 3 min. Subsequently, either collagen (1 μg/mL) or the Fenton reagent (50 μM FeSO_4_ + 2 mM H_2_O_2_) was added, and incubation proceeded for 5 min. Before ESR spectrometry, 100 μM DMPO was added to both the solutions. The ESR spectra were recorded using a quartz flat cell designed for aqueous solutions. The spectrometer was operated under the following conditions: power, 20 mW; frequency, 9.78 GHz; scan range, 100 G; and receiver gain, 5 × 10^4^. The modulation amplitude was 1G, the time constant was 164 ms, and scanning was performed for 42 s; each ESR spectrum obtained was the sum of four scans.

### 4.10. Measurement of Bleeding Time in Mouse Tail Vein

The bleeding time was measured through transection of the tails of male ICR mice. In brief, after 30 min of administering either 0.5 or 1.0 mg/kg Ir-11 intraperitoneally, the tails of mice were cut 3 mm from the tip. The tails were immediately placed into a tube filled with normal saline at 37 °C for measuring the bleeding time, which was recorded until the bleeding completely stopped. In the animal experiments, the method applied to the animal model conformed to the Guide for the Care and Use of Laboratory Animals (8th edition, 2011), and we received an affidavit of approval for the animal use protocol from Taipei Medical University (LAC-2016-0395).

### 4.11. ADP-Induced Acute Pulmonary Thromboembolism in Mice

A previously defined method was used to induce acute pulmonary [36]. Various doses of Ir-11 (0.5, 1.0 and 2.0 mg/kg) or 0.1% DMSO (all in 50 μL) were administered through intraperitoneal injection in mice. After 5 min, adenosine diphosphate (ADP, 0.7 mg/g) was injected into the tail vein. The mortality of mice in each group after injection was determined within 10 min.

### 4.12. Statistical Analysis 

The results are stated as means ± standard error of the means, beside the number of observations (*n*). The values of *n* refer to the number of experiments; each experiment was performed using different blood donors. The unpaired Student’s *t* test was used to determine the significance of differences between control and experimental mice. The differences between the groups in other experiments were assessed using analysis of variance (ANOVA). When the ANOVA results designated significant changes among group means, the groups were equated using the Student–Newman–Keuls method. A *p* value of <0.05 designated statistical significance. Statistical analyses were performed using SAS (version 9.2; SAS Inc., Cary, NC, USA).

## 5. Conclusions

The present, findings reveal that the novel Ir-11 compound powerfully inhibits platelet activation by inhibiting signaling pathways, such as the PLCγ2-PKC cascade, and subsequently suppressing Akt and MAPK activation. These alterations reduce granule secretion (i.e., ATP release, [Ca^2+^]i levels, and P-selectin expression) and ultimately inhibit platelet aggregation. However, additional studies are required to investigate the involvement of other unidentified mechanisms of the Ir-11-mediated inhibition of platelet activation. Ir-11 was intended to be used as a novel antitumor drug. However, it can be considered a new therapeutic agent that inhibits either arterial thrombosis or bidirectional cross talk between platelets and tumor cells.
